# Improving Maternal Survival in South Asia—What Can We Learn from Case Studies?

**DOI:** 10.3329/jhpn.v27i2.3324

**Published:** 2009-04

**Authors:** Barbara McPake, Marge Koblinsky

**Affiliations:** ^1^ Institute for International Health and Development, Queen Margaret University, Queen Margaret University Drive, Musselburgh, East Lothian, EH21 6UU, UK; ^2^ John Snow Inc., 1616 Ft Myer Drive, Arlington, Virginia 22205, USA

**Keywords:** Maternal health, Maternal mortality, Case studies, Asia, South, Bangladesh, India, Pakistan

## Abstract

Technical interventions for maternal healthcare are implemented through a dynamic social process. Peoples' behaviours—whether they be planners, managers, providers, or potential users—influence the outcomes. Given the complexity and unpredictability inherent in such dynamic processes, the proposed cause-and-effect relationships in any one context cannot be directly transferred to another. While this is true of all health services, its importance is magnified in maternal healthcare because of the need to involve multiple levels of the health system, multiple types of care providers from the highly skilled specialist to community-level volunteers, and multiple technical interventions, without the ability to measure significant change in the outcome, the maternal mortality ratio. Patterns can be followed however, in terms of outcomes in response to interventions. From these case studies of implementation of maternal health programmes across five states of India, Pakistan, and Bangladesh, some patterns stand out and seem to apply virtually everywhere (e.g. failure of systems to post staff in difficult areas) while others require more data to understand the observed patterns (e.g. response to financial incentives for improving maternal health systems; instituting available accessible safe blood). The patterns formed can provide guidance to programme managers as to what aspects of the process to track and micro-manage, to policy-makers as to what features of a context may particularly influence impacts of alternative maternal health strategies, and to governments more broadly as to the factors shaping dynamic responses that might themselves warrant intervention.

## INTRODUCTION

Mechanisms by which maternal health outcomes can be improved are social, not only technical. This point is evident in the case studies presented in this issue of the Journal—technical solutions are well-known, and it is the policy in countries where all the case studies have been done to make safe institutional delivery services available for all births and to provide a structure for referral to adequately-resourced emergency obstetric care (EmOC) in the case of complications. However, for reasons that are largely social—the behaviours of people as planners, managers, care providers, and service users—the reality fails to live up to the policy.

In summing up the case studies presented in this issue of the Journal and focusing on the social arrangements producing different maternal health outcomes in different settings, the reviewer is confronted with unpredictability. Technical solutions can be shown to work, or not to work. A timely blood transfusion will enable the recovery of a woman who would otherwise die in childbirth under given and known conditions. Social ‘solutions' of this kind are not available. Strategies that seek to improve access to timely blood transfusion will at best contribute to good outcomes in some cases but not in others, suggesting that lessons cannot be transferred unquestioningly from one context to another.

One difficulty in the application of lessons from one context to another is that the problem may be only superficially similar (high maternal mortality, for example) rather than similar in detail (failure to use an available maternal health system due to a specific combination of cultural and supply quality factors, for example). Depending on where a solution intervenes in the processes causing the problem, the differences at this more detailed level may be critical.

**Fig. 1.**
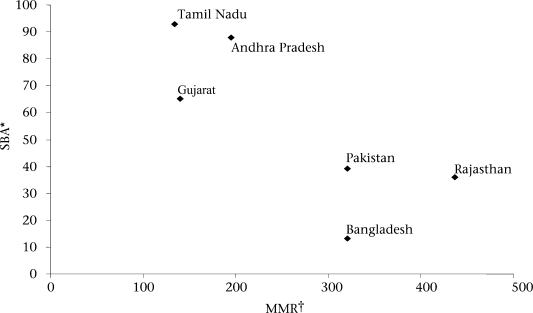
Maternal mortality and skilled birth attendence: selected South Asian countries and Indian states

Sources: (1–3, 4†–6); ∗SBA is % of births attended by a health professional; †MMR used the center point of the range of national estimates indicated; MMR=Maternal mortality ratio; SBA=Skilled birth attendant

The country/state-specific case studies included in this issue of the Journal exhibit similarities and differences at different stages of those processes. Figure 1 shows the position of each case study site on a scatter plot of maternal mortality ratio (MMR) and rates of skilled birth attendance and indicates wide divergence in these basic problem indicators. It highlights the gaps between Tamil Nadu, Andhra Pradesh, and Gujarat (MMRs less than 200, skilled attendance at birth above 60%) and Pakistan, Rajasthan, and Bangladesh (MMRs greater than 300, skilled attendance at birth below 40%).

Relatively better situations in Tamil Nadu, Andhra Pradesh, and Gujarat may imply that they have devised solutions that the sites of the other case studies could learn from. These case studies indicate significant progress in indicators (institutional deliveries increased from 20% in 1971 to 99% in 2008 in Tamil Nadu; skilled birth attendance increased from 66% in 1992 to 88% in 1999 in Andhra Pradesh and from 43% in 1992 to 65% in 2006 in Gujarat), and identifiable strategies may appear to have played at least some role in those improvements, for example, the Chiranjeevi Scheme in Gujarat.

There are obvious problems with a straight forward attribution of strategies used in the case studies to their relative maternal health performance. Reliable estimates of MMR are notoriously difficult, and the case studies of Gujarat (3) and India as a whole (7) document widely fluctuating rates, more likely to reflect errors of estimation than fluctuations in deaths themselves. Other case studies would caution against too direct an association between the use of skilled birth attendants (SBAs) and the MMR. In Rajasthan, for example, doubts were highlighted about the ultimate impact on the MMR of the *Janani Suraksha Yojana* (JSY) programme that appears to have dramatically increased the use of SBAs but perhaps neglected to assure the quality of care in the process (5). And in Tamil Nadu, while institutional deliveries increased from 65% in 1996 to 92% in 2003, the MMR increased from 131 in 1997/1998 to 167 in 2000/2001 (1)—which may reflect either improvements in data or lack of quality care.

In Gujarat, the Chiranjeevi Scheme appears to play at least some role in the explanation of the increased uptake of available maternal services (3). In Andhra Pradesh, it is less apparent which of the strategies identified, if any, might explain its better performance in maternal health (2). Indeed, the Andhra Pradesh case focuses elsewhere—on the broader social influences on maternal health, implicating on the one hand, the political priority that has been placed on maternal health compared to other state priorities and, on the other, the status of women in the state as a whole. This reminds that both current status of maternal health and recent changes to that may equally be derived from social factors and change in those as from changes in policy or new interventions.

One country where the decline in the MMR—of nearly 75% since the mid-1970s—is documented, is Bangladesh (6). While the MMR level in Bangladesh is still high compared to some Indian states, arguably it is the use of EmOC, safe menstrual regulation, and promotion of female education employed in Bangladesh that should be the focus of attention for policy transfer.

All these points caution against taking an overly deterministic view of what one country or state can teach another by exposing its case for analysis across borders. It is tempting to point to the need for more data, or more robust data, to reach more definitive conclusions. But realistic levels of availability of data, measurability constraints, and the number of available cases indicate that control of even the most obvious implicated factors, such as the status of women, nutritional factors, and quality of available services, could not be achieved. Moreover, the processes of determination of maternal health outcomes are likely to be ‘complex' (in the sense of complexity theory—for example, an apparently-insignificant detail can be amplified or an apparently-important factor dampened in its effects through a series of chain reactions), implying that controlling the most obvious implicated factors may not be relevant.

## CONCEPTUAL FRAMEWORK

**Fig. 2.**
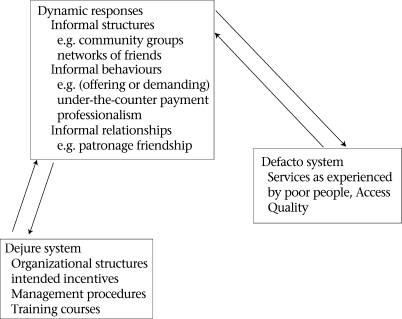
Dynamic responses model

Source: McPake B *et al.* 2006 (8)

In light of these difficulties in drawing policy implications usefully from comparative case material, a conceptual framework was developed to clarify the processes under comparison (Fig. 2) (8,9).

The framework distinguishes between the *dejure* health system (or the health system as it is designed and regulated) in which policy and intervention can be introduced and the *defacto* health system (the one that exists in practice) from which health outcomes emerge. In between there are a series of dynamic responses (in the sense that they arise from forces within and outside the health system and, in turn, exert forces of their own) that emphasize the social nature of a health system: they all relate to human behaviours. The critical feature of the framework is that it draws no direct link between the plan and experienced outcomes and, therefore, no necessary link between the provision of inputs and the organization and management of these inputs and the outcomes that emerge. The only health system that can be experienced by its users is the one that emerges from multiple human interactions. The framework focuses attention on how formal organizational structures, intended incentives, and management procedures interact with informal structures, behaviours, and relationships on the ground.

Given the complexity and unpredictability inherent in the processes involved in the dynamic responses, we have argued that proposed cause-and-effect relationships in one context cannot be directly transferred to another. Complexity theory does, however, suggest an alternative to a counsel of despair in relation to the use of comparative frameworks for applying evidence of research. It suggests that, rather than looking for data to provide rules to use in multiple contexts, we can rather use it to recognize patterns. These indicate sets of possible impacts of an attempt to intervene and can provide guidance to programme managers as to what aspects of process to track and micro-manage, to policy-makers as to what features of a context may particularly influence impacts of alternative maternal health strategies, or to government as a whole as to what factors shaping dynamic responses might themselves warrant intervention.

Given the social basis of the patterns, we may identify in reviewing maternal health evidence, the patterns evolve, plausibly even in response to their identification. Real human behaviours respond to financial and non-financial incentives and to cultural and professional value systems, all of which are always evolving, and to their prior experience with these. Hence, it will be helpful in reaching conclusions to heed the advice of Pawson and Tilley (10): “Never expect to know ‘what works' just keep trying to find out.”

## PATTERN RECOGNITION IN CASE STUDIES ON MATERNAL HEALTH

The following sections proceed in the attempt to recognize patterns across the case studies through the lens of the proposed conceptual framework and are organized across themes. Some patterns stand out and seem to apply virtually everywhere; for example, the failures of the systems that allocate staff to remote and difficult postings appear virtually universal, although Mavalankar has argued elsewhere that they are not evident in Sri Lanka, emphasizing that there are almost never wholly good or bad ways of doing things (11). Other patterns can be discerned only more tentatively, ultimately requiring collection of more data that seek to understand the mechanisms of effect that may be triggered in some instances and not in others. As section 2 below describes, various attempts to use financial incentives to motivate improved performance of the maternal health system seem to have had varying degrees of success. We may need to collect more data about how these different programmes have been implemented to build confidence in attributing the underlying patterns.

## Human resources

Common to all the case studies is a shortage of key personnel in the places they are needed to provide safe delivery services and EmOC. Table 1 shows the data provided.

**Table 1.** Measures of gap in human resources in the public sector (2,3,5,12)

Location-wise human resource	Ratio available: WHO requirement for maternal care	% filled posts (public sector)
Bangladesh		
Nurses		
Khulna–urban	3.09:1	107
Khulna–rural	0.37:1	97
Sylhet–urban	8.12:1	77
Sylhet–rural	0.18:1	54
Doctors		
Khulna–urban	12.25:1	84
Khulna–rural	1.25:1	60
Sylhet–urban	69:1∗	87
Sylhet–rural	0.75:1	34
Gujarat		
Multipurpose worker (PHC)		78
Female health assistant (PHC)		80
Male health assistant (PHC)		57
Doctor (PHC)		85
Obstetrician/gynaecologist (CHC)		3
Paediatrician (CHC)		2
Radiographer		37
Pharmacist		59
Laboratory technician		64
Nurse/midwife		53
Andhra Pradesh (PHCs)		
Medical officer		44
Trained SBAs (nurse-midwife/ANM)		33
Rajasthan	Ratio available: Government required	
Rural	0.93:1	
ANM at SC/PHC	0.77:1	
Doctor at PHC		
Tribal areas	1.71:1	
ANM at SC	0.68:1	

∗This number significantly affected by presence of a medical college in Sylhet city without which the ratio is 4.25:1 (12); ANM=Auxiliary Nurse Midwife; CHC=Community Health Centre; PHC=Primary Health Centre; SBAs=Skilled birth attendants; SC=Subcentre

No statistics available are a fully-satisfactory measure of the gap. The percentage of posts unfilled suggests that the two districts in Bangladesh (12) are broadly better staffed than either those of Gujarat (3) or Andhra Pradesh (2); however, the comparison depends on the levels at which numbers of posts have been set. The statistics indicate the extent to which absence of staff is due to failure to recruit or retain rather than failure to sanction the post (usually but not always an indication that the post has been budgeted for). Under this interpretation, difficulties in recruitment/retention are a greater problem in Gujarat and Andhra Pradesh, perhaps suggesting greater demand (e.g. more sanctioned posts per population level) than supply of qualified health professionals willing to work in the settings where posts are available.

In principle, the comparison with requirements of the World Health Organization (WHO) should be more robust, as these are statistics based on staff per 3,500 births but a variable share of time of posted staff is expected to be used for maternal care and probably a smaller share of a doctor's workload than of a nurse's. The data from Bangladesh indicate that the total urban workforce only need to apply a small percentage of their time to provide adequate maternal care but that more than the entire time available would have to be spent by nurses in rural areas on maternal care alone (12). This would also be true of rural doctors in Sylhet and in Khulna, they would have to spend an unrealistic 80% of their time on maternal services for an adequate service to be provided. The Rajasthan data indicate that the tribal areas have an adequate supply of auxiliary nurse-midwives (based on a government standard) but that other rural areas do not and that, in both types of rural area, there would be insufficient doctors for maternal care, even were they to spend 100% of their time on this work (5).

While equivalent data are not available for Pakistan, shortages of staff are also apparent (4). Situation analyses of EmOC in Sindh were carried out for 48 health facilities (district and subdistrict hospitals) in four districts in 1999 and for 200 health facilities in nine districts in 2000 (5). In principle, EmOC should be available in these facilities but, in practice, 60% referred patients to teaching hospitals for EmOC due to absence of staff—especially female staff—48% had no appropriate female staff at the time of the surveys. A full obstetric team was available in only 39% of facilities.

A common pattern across all the case studies is inequity in the distribution of available staff. Urban-rural disparity is illustrated by the data from Bangladesh (12) but will apply to some degree in all the cases. Despite what appears to be gross over-supply of doctors in urban areas, it is still the case that not all urban posts are filled, and the same applies to urban nurses in Sylhet whereas, in urban Khulna, the posts available constitute nearly three-fold the WHO recommendation for requirements of urban maternity services.

Clearly to some extent, this reflects choices made about the shape of the referral pyramid and perhaps the relative ease with which people can reach urban areas in densely-populated Bangladesh. As it is documented in all cases in which it is explored, the situation also clearly reflects health workers' choice of location and the failure of the universally-used posting system to place health workers where they are needed. In other words, dynamic responses among health workers and administrators of the posting system interfere and leave gaps in the available human resources in the system.

The posting system exhibits the discredited features of similar central planning approaches in other contexts—it seeks to direct people irrespective of their preferences and is liable to governance and stewardship failures. Those few willing to serve in remote or difficult postings, or unable to escape them, encounter the triple burdens of difficult working conditions, an under-staffed environment that makes those conditions more difficult still, and (often) disadvantage in career progression. The result is not just scarcity of skilled staff, but frequently also demotivation and demoralization that can manifest as poor-quality care.

In some case studies, measures have been put in place that seek to interfere in this pattern. The Chiranjeevi programme seeks to enlist the more numerous private-sector care providers to maternal healthcare provision in remote and difficult areas (3), based on an understanding that it will require incentives and adequate finance to do so, and also in Gujarat, the ‘walk-in' recruitment system indicates an openness to finding other ways of doing things. Similarly, in Rajasthan (5) and Tamil Nadu (1), contracted staff who have proved in other settings less able to shift their employment to another location have been a component of the strategies adopted. For example, the Capacity Project's Emergency Hire programme in Kenya has effectively recruited nurses and other health professionals through contracts tied to specific rural locations and succeeded in significantly increasing available health staff in rural areas in the context of a much-abused posting system that creates significant urban-rural inequity of staffing (http://www.capacityproject.org/index.php?option=com_content&task=view&id=133&Itemid=108). In Andhra Pradesh, the scheme of female health volunteers seeks to replace missing public-health workers with volunteers, and the RCH Health Melas also seek to plug resulting gaps in services (2). In Tamil Nadu, the incentive of securing postgraduate training places has attracted young doctors to what would otherwise be unattractive positions (1).

Mavalankar has commented elsewhere: “Overall, it seems that central and state governments are unable to or unwilling to take up major governance issues … (of a transparent and well implemented posting and transfer system) … which hamper delivery of services in rural areas” (11).

In line with this, there seems little appetite for a complete reconsideration of posting as the principal means of distributing available health staff, despite evidence that when incentives and adequate finance are combined, resources can be effectively marshalled (for example in the Chiranjeevi Scheme). Some explanation appears to lie in the entrenched interests that gain from its continuation.

A further human resources-related pattern discernible in at least two case studies is a constellation of factors confounding more effective management of the health system. One set of factors concerns the lack of management training among those given management responsibilities. Most accounts of the shortage of human resource fail to consider the scarcity of health managers, and this gap tends also to be overlooked when strategies are devised to tackle the shortage of human resource. It has been argued that, globally, shortages in the area of managerial support for health systems may be more acute even than those of clinical workers (13).

The issue is particularly highlighted in the Gujarat case study (3) where a factor identified as potentially responsible is that of the hierarchical position of obstetricians and surgeons in the maternal healthcare system. Tension has been observed in other settings between the hierarchical power assumed by medical and surgical disciplines and the failure to either allow those from other backgrounds the authority to manage, or to accord sufficient weight to effective management in the career paths of doctors and surgeons. It remains to be seen whether Gujarat's initiative to create ad-hoc positions of District Programme Coordinators with health-management backgrounds (3), or Tamil Nadu's appointment of Deputy Directors with postgraduate qualifications in public health (1) will succeed but similar initiatives elsewhere have sometimes foundered on the inability of those put in such positions to wrest sufficient authority to direct resources (14,15).

Management capacity could be acquired through practice of management but, too often, managers are rotated quickly in and out of managerial and clinical roles for that to occur.

## Financing and incentives

Issues of financing and incentives have already arisen in the context of human resources but have a more widespread influence in the patterns emerging from the case studies. Formal incentives may be set to reward specific achievements (for example, the bonus paid for night-time delivery in Rajasthan) and, therefore, to exert influence over dynamic responses on the basis that it is difficult to regulate effort directly and easier to encourage it by rewarding outcome.

Nevertheless, incentives themselves can undergo a reshaping through dynamic responses so the formal incentives do not necessarily apply. For example, bonuses intended to reward a particular achievement may, in practice, be allocated to reward something different, or those seeking rewards can misrepresent the achievement. Hence, managing incentives requires capacities of the health system that may not differ greatly from those capacities involved in managing staff performance more directly.

Attempts to manipulate incentives can apply to either demand- or supply-sides of maternal health, i.e. care providers can be offered incentives to provide more care, and potential users can be offered incentives to use more services. The case studies highlight a number of apparently-successful initiatives to manipulate incentives of both types, most notably in Gujarat's Chiranjeevi Scheme (3) which operates on both demand- and supply-sides.

The Chiranjeevi Scheme's incentives are very carefully designed. It is clearly no accident, for example, that the payment to private practitioners is for a package of 100 deliveries rather than a price per delivery and that the scheme designers were seeking to control incentives to inflate the level of complications claimed and probably, in particular, the number of caesarean sections carried out. By estimating a reasonable level of these, the scheme's designers aimed to ensure that sufficient resources are available to cover the costs of genuinely complicated cases while offering no additional funding for each complicated case claimed.

The scheme appears to have been very successful in attracting the private-sector service providers to join it and in enabling a significant number of deliveries—prices would appear to have been set at levels capable of attracting the involvement of the private sector. It also appears to have been successful in achieving quite low rates of caesarean and complicated deliveries (about 12% overall (3), although rather higher at about 23% in Dahod district in which the more detailed study took place 16). All these suggest that the pricing scheme set has not been significantly distorted in its implementation.

Nevertheless, a number of dynamic responses seem to have distorted some aspects of the scheme. Bhat *et al*. showed that there was little difference in the socioeconomic status of those included and excluded from the scheme, which was surely not what was intended (16). It is not clear what kind of dynamic responses may explain that, as such responses could either arise in the system by which people achieve the ‘below poverty-line' (BPL) formal status (cards) or in the system by which those with and without such status access the scheme itself. Alternatively, the outcome may simply reflect variation in the extent to which knowledge about the scheme has spread; for example, those accessing antenatal services might be most likely to hear about it, cancelling out the scheme's bias towards the poorest. Although the users are largely ‘poor', 6% have access to it despite having an income level of above Rs 12,000 per month which would appear to locate them in the upper 10% of the income distribution in the district. The fact that this is still a very low level of income, classified as poverty by international agencies, suggests that the attempt to target in such districts as Dahod may be largely futile. The scheme might be more successful if it were to recognize the poverty of the vast majority and offer universal access.

Other signs of dynamic human intervention are evidenced in other issues emerging from the scheme. Clients reported not receiving food (93%), having to pay for medicines (80%), not receiving payment for transport (relatively rare), and nurses asking for money (relatively rare). These issues highlight the difficulty in monitoring the receipt of services that clients are entitled to and, therefore, of penalizing failure to deliver the full package. As the scheme matures, it is hoped that local knowledge of which providers offer the best package of care under the Chiranjeevi Scheme would put pressure on providers to deliver as contracted; however, this depends on there being a sufficient number of care providers that any given user has a choice. It is not clear that this is yet the case.

The Chiranjeevi Scheme is not the only example within the case studies of attempt to pay directly for safe delivery. Using additional incentives for public-sector workers implies that receipt of a salary is quite widely, if implicitly, acknowledged as insufficient incentive to ensure adequate attention to this service. Targeted incentives may be more effective, or at least more cost-effective than the suggested generalized salary increases needed by the Millennium Development Project, but which are less evidenced across the case studies. The use of contracted staff in Tamil Nadu (1) and Rajasthan (5) and the payment mechanism employed to manage women health volunteers in Andhra Pradesh (2) may circumvent some constraints imposed by the normal conditions of public service.

The JSY programme, operating in Indian states, including Andhra Pradesh (2) and Rajasthan (5), seeks to apply similar principles but, in this case, focused on encouraging women to take up available services. In Andhra Pradesh, this has been supplemented by a free-bus pass scheme to further protect users against transport expenses (2). There, it has been targeted on those with BPL status and will, therefore, be equally affected by any distortions of policy that arise in the allocation of BPL cards (if those exist). However, in Rajasthan, the programme has been extended to cover all pregnant women (5), perhaps in response to those kinds of problems, or perhaps in recognition of the issue apparent in Dahod— that if the vast majority of the population is ‘poor', elaborate mechanisms to filter the more poor from the less poor may be neither warranted nor workable.

As the Chiranjeevi Scheme, it appears that the JSY scheme has had the desired immediate and direct effect at least in Rajasthan: numbers taking up institutional delivery have increased significantly—a 10-fold increase in uptake between its first and second years of operation, for example, and, overall, large improvements in institutional and SBAs-assisted births since 1992 (5). The principal caution expressed is that the quality of care may not support the translation of institutional deliveries into safe deliveries. A similar problem seems to have arisen from efforts to increase the rate of institutional deliveries in Andhra Pradesh (2).

Bangladesh appears to have regulations in place, making it difficult to change the formal incentive programme there; so, for example, no incentives for serving in remote or difficult postings seem feasible to implement (6). However, the maternal health voucher scheme, introduced in 2007, appears to have circumvented such regulation, and, perhaps, further innovation will prove possible. This scheme provides incentives to both care providers and mothers to provide and take up safe delivery services. It is too early to judge the effects of this scheme (6).

The pattern apparent in the case studies is that financial incentives do motivate changes in behaviour. However, mechanisms to monitor the application of incentives and the provision of services rewarded are often critical and needed to prevent ‘perverse' incentives from arising, for example, to report activity which has not taken place (the suggestion in Rajasthan that health staff report deliveries to have occurred at night to claim additional allowances would furnish one example (5), or to deliver an indicator (for example, births in an institution) without delivering the outcome it is supposed to indicate (safe delivery).

It is equally important to recognize that the absence of positive incentives can leave negative ones to rule. For example, as has already been discussed, poor salaries, lack of promotion opportunities and transparency in posting, and transfer and promotion have been indicated in the case studies, including Bangladesh, as undermining service provision across the board. Moving beyond the ‘command and control' mechanisms requires the development of new health-system capacities, and it appears that the Indian states are recognizing this and beginning to develop the relevant ones.

Among these are the capacities to learn from pilot implementation and scale up in the light of that learning. Tamil Nadu's use of Theni as a district piloting ground (1) and Gujarat's five-district trial of the Chiranjeevi Scheme (3) provide examples. In contrast, it does not appear that Bangladesh completed the evaluation of the maternal health voucher scheme pilot in three districts and is seeking to scale up without an adequate evidence base.

Not all incentives are financial, and Tamil Nadu's use of specialist training places as a reward for rural service (1) is an example of innovative use of gifts the public system has at its disposal in place of financial bonuses with budgetary implications. While detailed examination of this system has not been undertaken, it is clear that dynamic responses are equally capable of intervening in non-financial and financial domains, for example, by allocating training places other than according to the policy. Hence, non-financial incentives equally need effective systems to be established to monitor and control any abuses of systems that might emerge.

## Organizational structure

India, Bangladesh, and Pakistan are all affected by the division of responsibilities for maternal health and family planning. Few benefits are identified as flowing from this division.

In Bangladesh, there are two separate wings of the Ministry of Health and Family Welfare, and although there was attempt of unification after 1997, this was abandoned after 2003, largely because of the practical difficulties of re-allocating authorities within the system (17). The division implies a dual chain of authority over field workers and direct care providers and that reporting and information systems are separated. The principal problem identified as associated with this division is lack of coordination. Field workers and care providers are confronted with overlapping responsibilities for the same populations, and the results appear to be a duplication of efforts in some places, gaps in services in others, and overall inefficiency of resource-use.

The Bangladesh case study charts several attempts to overcome these problems, including the attempt to unify the two wings (6). When fieldworkers of the family-planning wing were asked and trained to take on maternal health responsibilities, the activity failed to fully materialize and their focus drifted back to family planning. Other attempts to forge more coordinated activity seem similarly to have foundered.

There have similarly been several attempts to merge the two separate ministries in Pakistan (where the division is between Health and Population Welfare), recognizing the inefficiencies of overlaps and failure of coordination that the split implies. As in Bangladesh, these have failed, likely for similar reasons.

The similar division in India is between the Department of Family Welfare and Department of Health (7). This divorces delivery of healthcare from research and training, and coordination problems also arise. Also similar to the Bangladesh case is the relative strength of the family-planning agenda compared to the maternal health agenda, and although the delivery of the two is better integrated in India, neither type of arrangement seems to protect maternal health from neglect in the context of significant national government political commitment to family planning backed by both national and international resources.

Field workers in this system have been confronted by a job description with multiple objectives. It is well-understood from a theoretical perspective (18) that, under these circumstances, strengthening the measurement and monitoring of outputs will favour measurable, quantifiable activities compared to ‘softer', more difficult to measure outcomes, such as quality of care. The greater measurability of family-planning outputs, unconstrainted by a shared responsibility for maternal health outcomes has clearly resulted in the neglect of maternal health duties in some cases [in Bangladesh (6) and in Gujarat, India (3)]. This problem is only partially tackled by the renewed emphasis on maternal health reflected in and effected by Millennium Development Goal 5. The inherent greater measurement difficulties undermine the maternal healthcare effort. Hence, efforts to improve measurement (see below) are important for improving the capacity to manage field workers and for evaluation and advocacy.

## Role of the private sector

In most case studies, little information is presented about the private sector, presumably reflecting the limited availability of data and the limited emphasis on the private sector in policy and strategy in relation to maternal health. Arguably, and perhaps to a varying extent across the cases, the private sector is not supposed to be there—a universal public system has been planned, and this still affects the conceptualizing of the health system, despite the growth of understanding of the importance of the private sector in health, generally, estimated to account for 71%, 81%, and 83% of health expenditure in Bangladesh, India, and Pakistan respectively (WHOSIS, http://www.who.int/whosis/database/core/core_select.cfm—all estimates for 2005).

A significant share of maternal health provision takes place in the private sector. The Rajasthan case study estimates that 28% of deliveries occur in private hospitals there (5) whereas, in Tamil Nadu, the most recent estimate is 36% in private nursing homes (1). There are numerous private healthcare providers in Pakistan: 20,000 general practitioner clinics, 340 dispensaries, 200 small maternity homes or MCH centres, and 500 low-to-medium-bed hospitals (4). The World Bank data suggest that delivery in the private system is most common in India, averaging 17.4% and somewhat less so in Pakistan (6.2%) and Bangladesh (3.2%), although the data must be treated with some caution in assessing relative levels owing to the wide divergence in dates for which data are available (Table 2).

**Table 2.** Delivery in the private sector (%)

Country and period/quintile	1 (poorest)	2	3	4	5 (richest)	Average
Bangladesh 2004	0.2	0.7	0.9	3.5	13.6	3.2
India 1998/1999	3.5	6.6	13.6	24.7	50.8	17.4
Pakistan 1990/1991	0.4	0.3	1.8	5.7	23.5	6.2

Source: World Bank health, nutrition and population database (http://siteresources.worldbank.org/EXTHNPSTATS/Resources/3237117-1170098293815/3387570-1208958254373/Antenatal_and_Delivery_Care.xls)

Formal private-sector provision may be of better quality than informal but is largely still considered poor outside highly visible exceptions in urban areas. For example, the private sector in Pakistan was popular because anticipated quality was good, but more than 50% of families reported that the quality of services at private hospitals did not match expectations, and 70% perceived some mismanagement of their cases by private providers (4). The narratives of critical incidents in Jessore and Sylhet highlight some potential patterns in describing the differences among formal private, informal private and public care in Bangladesh (19). For example, the narratives identify problems of waiting times in public care that result in decisions to try elsewhere; in informal private care, practices unlikely to promote safe motherhood abound such as the recourse to ‘blessed water' to control convulsions, and in formal private care, the inability to cope with a health crisis with referral of patients back to the public sector with potentially harmful implications of delayed effective care.

Private-sector care is accessed to a greater extent by richer than poorer citizens in all three countries (Table 2).

Traditional birth attendants (TBAs) who are private providers are the most numerous maternal healthcare providers—estimated to number 3,213 in Udaipur district, Rajasthan, which can be compared with 136 traditional practitioners and 122 allopathic MBBS or MD qualified doctors (5). The earliest type of intervention seeking to tap the unused capacity of the private sector was the prevalent strategy of training TBAs advocated by the WHO continuously between 1978 and 1997. The mixed experience with this intervention may provide lessons for more currently-emphasized strategies to achieve public-private mix. The strategy has been considered, on the basis of cross-country global evidence of unproven impact on maternal mortality (20) but generally, it has not been very systematically evaluated [as is reported in Rajasthan (5)]. As with other types of intervention that involve attempts to change behaviours of individuals (in this case, largely TBAs' hygiene procedures and referral decisions), it is unlikely that clear and consistent outcomes can be measured at a global level. Rather, local studies need to seek to understand how the strategy works in context with particular categories of TBAs and TBA-client pairs, and therefore, where and under what conditions it may be capable of playing a positive role. In Bangladesh, it is suggested that TBAs and trained TBAs may have contributed to the decline in maternal mortality, although one study of the effect of training in hygiene only, suggested that it was unlikely to have been sufficient to have affected maternal morbidity or mortality (21).

In Bangladesh, the most numerous providers were counted as traditional practitioners (‘*kobiraj*', ‘*totka*', herbalists, and faith healers) with a density of 64.2 per 10,000 population, followed by TBAs (density 33.2) and ‘village doctors'—also popularly known as ‘quacks'—who practise allopathic medicine but without training or registration (density 12.5) (22). The importance of village doctors has been identified by a number of the case studies, including but not restricted to Bangladesh. The narratives from Jessore and Sylhet identify specific roles that these care providers play, including providing specific interventions, such as oxytocin injections that TBAs do not (12). The same study found that these doctors are often the first resort on signs of difficulties with a birth on the grounds that they are experienced in administering a number of treatments in which people have confidence and that they are available and near so that treatment can commence more quickly. In this and another study in Bangladesh, it was found that these doctors were often instrumental in the decision to abandon attempts to give birth at home, or with TBAs, and to seek formal care instead (23).

Additionally, the private sector plays a particularly important role in the provision of abortion services, which, in turn, play a particularly important role in maternal mortality. The Rajasthan case study provides the most detailed account of issues relating to abortion services, the conclusions from which may well apply in other cases under review (5). It documents bureaucratic obstruction to the certification of formal care providers that is likely to account for difficulties in access of people to certified facilities—only 0.6 per 100,000 people and highly skewed to particular districts. It documents features of formal provision that are inconsistent with making the maximum contribution to reduction in mortality, such as the tendency of government providers to charge for this service despite its formal free status, and the further tendency to particularly exploit the most vulnerable women in this way. This is likely to drive women and particularly the most vulnerable to the informal care providers who deliver 67% of abortion services in Rajasthan but whose quality of care is documented to consist of a number of practices with a high chance of being injurious to women. In particular, their use of harmful invasive techniques is evident from media reports of women dying from unsafe abortion, verbal autopsy studies, and survey data of the Sample Registration System that show that 10% of maternal deaths are due to septic abortion (24).

In Tamil Nadu, the rate of abortion is thought to have been increasing (1), and it is significantly higher in this state than in India as a whole (rates of 4.3% and 7.7% of all pregnancies compared to 1.3% and 4.5% of induced and spontaneous abortions respectively). One factor identified as encouraging use of the private sector was the tendency of the public sector to carry out permanent sterilization as part of the procedure. Private nursing-homes provide an alternative for richer women but it is likely that poorer women turn to care providers of unsafe procedures in the informal private sector. Six percent of deaths were deemed associated with denial of access to safe abortion, and measures are being taken to improve access to safe abortion.

Bangladesh documents a decline, over time, in the rate of abortion-related mortality (25), and it would be interesting to know more about the extent of the involvement of the private sector and its potential role in that. Improved government provision in relation to menstrual regulation and services to treat complications arising from induced abortion, combined with the better availability of family-planning services, may wholly explain this reduced mortality, however.

The private sector also plays an important role in offering alternative opportunities to those who might be providing safe delivery and related services in the public sector or elsewhere. The issues arising for both location-choices and absence from duty stations from the parallel private practices of those employed in the public sector have been highlighted in Bangladesh (6). Nevertheless, on the limited basis of the comparison between the two districts studied in this respect only, the combination of good private-practice opportunities, consequent popularity as a location-choice and absenteeism associated with parallel private practice appears associated with better outcomes than the reverse situation.

The growth of the private sector documented in Gujarat (3), Maharashtra (26), Rajasthan (5), and India as a whole (7) suggests that more attention to documenting the role played by formal and informal private care providers should be recommended as a priority, as a means to ensure that maternal health strategy remains relevant to realities and to capitalize on opportunities that arise from this growth. Strategies documented in all the Indian case studies indicate that these are beginning to be recognized by policy-makers.

## Blood

As reported by the Rajasthan case study (5), the leading causes of maternal mortality in Asia are haemorrhage (30.8% of all deaths) and anaemia (12.8%), both of which may give rise to the need for blood transfusion as a lifesaving intervention.

Inadequate access to blood for transfusion appears to be significantly implicated in maternal mortality, and this issue assumes greater importance as the proportion of births that take place in institutions increases and the most proximate causes of most deaths are not failure to seek appropriate care or reach an appropriate facility in time. In Bangladesh, it was found that, although not universal, appropriate reactions to postpartum haemorrhage were more common than not and that blood may be available at the level of the Upazilla Health Complex [for example (19), in Fig. 1].

The case studies document a common set of problems that undermine adequate access to blood for transfusion. There are inadequate numbers of blood-banks compared to the recommendations of WHO in those cases that reported on this. For example, compared to the WHO's recommendation of one blood-bank for every 100,000-120,000 people, Gujarat has one blood-bank for every 313,000 people (3) and Bangladesh one blood-bank for every 523,000 people (6). The case studies document variously: inadequate funds and government commitment to safe blood services, a shortage of donors, particular issues of access in rural areas, problems with quality control, lack of appropriate linkages between blood-banks and health service providers, and problems with rational use of blood.

A number of strategies have succeeded in overcoming these problems in some places. Maharashtra has used a range of approaches to encourage more blood donation using popular media and information, education, and communication (IEC) materials (26), and in both Maharashtra and Gujarat, numbers of blood donors have been increasing and the need to use less reliable mechanisms reducing as a result (26). Another important factor in these increases appears to have been the greater use of blood-donation camps.

A number of Indian states have recognized that the recommendations of WHO of the ratio of blood-banks to population will not be achieved in the short run and that facilities need to source blood without necessarily accessing full-scale blood-banks. Gujarat and Rajasthan have both identified the strategy of setting up blood-storage facilities at the facility level to store blood sourced from inaccessible blood-banks when it is needed (5,26). Measures allowing for more ad-hoc responses have also been put in place, including the use of ‘replacement donors', relatives of blood recipients, and the maintenance of lists of willing local donors in District Departments of Health. These kinds of strategies allow transfusions to take place despite the lack of adequate blood-banking facilities. Thirty-five of the 41 facilities in Bangladesh that were studied had transfused blood in the last month despite most having no facilities for blood-grouping and matching, collection-bags, or storage facilities (6).

Measures to try to improve the national and state responses to the need for safe blood supplies operate in an increasingly-diversified market for blood, such that, of 2,063 blood-banks in India as a whole, 980 are private and 257 are voluntary (26). Although the private sector fills a gap, it is still estimated that only 40-60% of the need for blood can be met using the total available and that the inefficient use of blood supplies lowers that estimate further. The resulting fragmentation of the blood supply causes facilities and patients and their relatives to waste time and resources in search of the appropriate blood product.

The strategies mentioned to address the problems of blood supply have generally not been evaluated but a pattern may be discerned of a slowly- improving situation, at least in Maharashtra and Gujarat (26), suggestive of approaches that can both increase overall supplies and integrate blood-bank systems around a system of predominantly voluntary donation. If these strategies can develop further and be adapted for use in the other case-study settings, they would seem to offer scope for driving reductions in maternal mortality across the sites.

## Evaluation

An implicit pattern across the case studies is an absence of routinely-collected data about key features of maternal healthcare provision and its outcomes. Most critical are the difficulties of measuring MMRs and the consequent ambiguity of other potential measures of progress (e.g. process indicators, such as use of SBAs). Given this basic problem, further key analyses, such as accurate breakdown of direct causes (current estimates are biased by the greater information availability about hospital-based events), are absent.

An important tool for filling some among these gaps is the verbal autopsy which can shed light on the social and biological factors implicated in maternal deaths. Tamil Nadu (1), Andhra Pradesh (2), Rajasthan (26), and Pakistan (4) all report the recent use of this tool for this purpose, and the differences highlighted by analysis of the results seem to provide insights into the relative strengths of the respective health systems. For example, in Andhra Pradesh, 20% of women died on the way to hospital (2) whereas, in Pakistan (4), the rate was 2%. The difference seems likely to be explained by the contribution of Pakistan's Edhi Foundation, an NGO that provides ambulance transport and whose model of operation could provide insights for others.

Useful as these measures are, an understanding of the health-system factors implicated in outcomes requires a monitoring and evaluation process that digs much deeper into the processes that lead up to maternal deaths and other bad outcomes. In principle, there are layers of implicated factors: those involved in the take up of services offered, such as those determining access; those involved in the quality of services received such as the systems that ensure that resources are available and incentives to motivate their appropriate use; and those involved in shaping those systems such as the policies that are in place, and the resources that are made available to support the implementation of those policies.

The critical gaps in information for monitoring and evaluation are those involved in understanding the extent to which things that have been regulated or legislated exist in practice: in the terms of Figure 2, the detail of the *de facto* system. While factors that determine whether services once made available are used, are equally important in determining the ultimate impact of programmes, they are not so clearly the responsibility of programmes.

In all the case studies, availability of staff and presence or absence of critical inputs to safe delivery, such as blood and basic equipment and supplies, could only be determined by an ad-hoc survey. There is no routine reporting system for these. Routine monitoring of the gaps in the basic infrastructure would allow for regular review of why gaps are emerging: whether resources are not budgeted, whether budgeted resources are not reaching the levels of the system required, whether there is a leakage of resources that have been provided, whether posted staff members have renegotiated their posts or are routinely absent, and whether it is proving difficult to recruit or retain health workers of particular cadres. The routine tracking of gaps in provision back through layers of factors implicated in these gaps would provide basic information for the effective management of maternal health programmes that could be much more sensitive to emerging and changing realities than any manager can be under current information constraints.

## CONCLUSIONS

The South Asian story is one of impressive achievements and improvements over the most recent decades. Where the time-trend data have been presented, these show great increases in the proportion of births taking place in institutions or with skilled attendants, and overall trends of remarkable declines in MMR. Although, in some cases, the MMR trend appears erratic, this is more likely the effect of measurement difficulty—overall declines over longer periods are consistently downwards.

These improvements have occurred despite the widespread documented difficulties of achieving adequate, functional and accessible safe delivery services. Where the situation of frontline service provision has been studied in detail, it consistently highlights non-availability or non-functioning critical components of a safe-delivery service, including lack of access to blood, non-availability of basic equipment, and absence of the necessary skilled personnel for both basic and emergency obstetric care. The widespread use of the informal private sector and the formal private system, despite its financial inaccessibility, reflects the failure of the public system to offer an acceptable service. Although failures to provide adequate care in the private system are also well-documented, these have not been the focus of the case studies in this issue of the Journal.

This combination of observations presents, superficially at least, a puzzle and one that has been explicitly analyzed by the Bangladesh paper (25) that exhaustively considers alternative theories of change behind the decline in MMR. That paper is unable to single out one dominant explanation, and it is likely that, in the other cases too, a combination of factors has been responsible. While the services available in present-day public facilities exhibit clear defects compared to what is desirable, those available 10 or 20 years ago have not been adequately documented to enable a valid comparison. The case studies of responses to postpartum haemorrhage and eclampsia verify that the public- health system is able to intervene at critical moments and effect better outcomes, for all its documented shortcomings.

A few case studies have focused in depth on the larger social changes that may also provide some explanation. It is widely understood that economic and social change has been rapid in South Asia, perhaps most so in India where real per-capita income growth averaged 2.6% between 1970 and 2004 (27) and the status of women is in flux, improving in at least some sections of society (28). Bangladesh has also seen positive economic and social trends in recent years with GNP per-capita having grown from US$ 217 in 1995 to US$ 445 in 2005, and women achieving a higher rate of secondary school enrollment than men by 2002-2003 (WHO country health-system profile—http://www.searo.who.int/en/Section313/Section1515_6122.htm).

The initiatives documented in the case studies in this issue of the Journal and suggested to have played a specific role at least in increasing the use of institutional delivery assistance, such as Chiranjeevi Scheme and JSY, are notably separated from the main health system and are separately labelled as ‘schemes' and ‘programmes' and budgeted additional to the running of the mainstream system. As such, these testify to the inventiveness of key individuals who have been able to lobby and secure funding for discrete activities and then to make sure that these are implemented effectively.

However, these also testify to the relative difficulty of more wholesale change within the public system. In effect, these programmes recognize that there are opportunities to benefit from unused capacity in the private sector, that public-service human-resource management works poorly, and greater flexibility and the use of incentives are required to ensure that priority activity happens and that intended beneficiaries of programmes may need support in accessing care and cannot be assumed passive recipients. In contrast, these insights appear to be lacking in the mainstream health system where pervasive failures are repeatedly documented. There is resistance to adopt these principles for routine business rather than for ad-hoc programmes, and this resistance results in wasted resources—like the presence of only one half of the gynaecologist-anaesthetist pair required to provide EmOC in Bangladesh (6)—and potentially, a fragili-ty of the sustainability of the special programmes that may be overly dependent on key individuals and on ad-hoc approvals for continuation of funding.

In a recent novel, Mary Lawson describes the (fictional) response of early 20th century rural Canada to the problem of access to medical care by remote communities:

“Ian's grandfather had been Struan's first resident doctor and, when he'd answered the Doctor Wanted advertisement they'd put in a Toronto medical journal, the grateful townspeople built him a house just a block west of Main Street ….

… as much as anything else, the building of the house had been a statement of faith on the part of the people of Struan. Until then they'd had to go to New Liskeard if they required a doctor, and if you needed medical help badly enough to make the journey to New Liskeard, the odds were that you were in no state to make the journey. Getting their own doctor was a sign that the town had arrived. In the brief interval between applying the final coat of paint and the arrival of Dr Christopherson, the people of Struan found excuses to walk past the house and admire it. You looked at that house and you thought, this is no fly-by-night northern settlement sprung up around a sawmill; any town that can afford to build its doctor a house like this is here to stay.” (Lawson M. The other side of the bridge. London; Vintage Books, 2007)

This account seems to identify things missing in the bureaucratic posting systems used as the main mechanisms to treat the same problem universally across the case-study settings. Perhaps most critically, it allowed for the person attracted to such a remote posting to identify himself rather than someone who would almost certainly find it a deprivation to be commanded to take up the post and on the community to engineer their own solution and take pride in the outcome.

In the modern global economy and in the South Asian context, approaches to achieve a better distribution of health staff will be different, but will likely apply the same mechanisms (community leadership and self selection). Nevertheless, it is apparent that, despite recognizing the requirements of a functional system, a wholesale change in that direction does not occur. Clues as to why might be found in Bangladesh's difficulties in trying to make the wholesale change of integration of the department of health and family welfare and the conflicts that identified with vested interests in the status quo. This echoes experience with more wholesale health-sector reform in multiple contexts across high- and low- income countries and widespread geographical regions. Even when a fundamental change to system operation is legislated, it is often resisted and subverted at implementation and tends not to achieve its intended effects.

All this implies that effective strategies will involve not only state and national-level policy change—as far as is feasible reflecting the principles that are understood to support better functioning systems—but also capacitating local managers to recognize and work with the dynamic responses between such policy and its outcomes. Such capacitating involves not just equipping with relevant skills but also working at successively higher levels of the system to support the delegation of the appropriate authorities to allow those skills to be put to use. Clearly, this is not an overnight ‘fix' but the objective of long-term strategies to achieve social development in all its dimensions, including maternal health.
